# Systematic Review of the Common Pathophysiological Mechanisms in COVID-19 and Neurodegeneration: The Role of Bioactive Compounds and Natural Antioxidants

**DOI:** 10.3390/cells11081298

**Published:** 2022-04-11

**Authors:** Kyonghwan Choe, Hyun Young Park, Muhammad Ikram, Hyeon Jin Lee, Tae Ju Park, Rahat Ullah, Myeong Ok Kim

**Affiliations:** 1 Division of Life Science and Applied Life Science (BK21 FOUR), College of Natural Sciences, Gyeongsang National University, Jinju 52828, Korea; k.choe@maastrichtuniversity.nl (K.C.); qazafi417@gmail.com (M.I.); dlguswls363@naver.com (H.J.L.); rahatullah1414@gmail.com (R.U.); 2 Department of Psychiatry and Neuropsychology, School for Mental Health and Neuroscience (MHeNs), Maastricht University, 6229 ER Maastricht, The Netherlands; 3 Department of Pediatrics, Maastricht University Medical Center, 6202 AZ Maastricht, The Netherlands; hailey.park@maastrichtuniversity.nl; 4 Department of Psychiatry and Neuropsychology, School for Mental Health and Neuroscience (MHeNS), Maastricht Medical Center, 6229 ER Maastricht, The Netherlands; 5 Haemato-Oncology/Systems Medicine Group, Paul O’Gorman Leukaemia Research Centre, Institute of Cancer Sciences, College of Medical, Veterinary & Life Sciences (MVLS), University of Glasgow, Glasgow G12 0ZD, UK; t.park.1@research.gla.ac.uk; 6 Alz-Dementia Korea Co., Jinju 52828, Korea

**Keywords:** COVID-19 and neuroinflammation, pathological bases and therapies, inflammation, cytokine, neurodegeneration

## Abstract

The novel coronavirus (2019-nCoVCOVID-19) belongs to the Beta coronavirus family, which contains MERS-CoV (Middle East respiratory syndrome coronavirus) and SARS-CoV (severe acute respiratory syndrome coronavirus). SARS-CoV-2 activates the innate immune system, thereby activating the inflammatory mechanism, causing the release of inflammatory cytokines. Moreover, it has been suggested that COVID-19 may penetrate the central nervous system, and release inflammatory cytokines in the brains, inducing neuroinflammation and neurodegeneration. Several links connect COVID-19 with Alzheimer’s disease (AD), such as elevated oxidative stress, uncontrolled release of the inflammatory cytokines, and mitochondrial apoptosis. There are severe concerns that excessive immune cell activation in COVID-19 may aggravate the neurodegeneration and amyloid-beta pathology of AD. Here, we have collected the evidence, showing the links between the two diseases. The focus has been made to collect the information on the activation of the inflammation, its contributors, and shared therapeutic targets. Furthermore, we have given future perspectives, research gaps, and overlapping pathological bases of the two diseases. Lastly, we have given the short touch to the drugs that have equally shown rescuing effects against both diseases. Although there is limited information available regarding the exact links between COVID-19 and neuroinflammation, we have insight into the pathological contributors of the diseases. Based on the shared pathological features and therapeutic targets, we hypothesize that the activation of the immune system may induce neurological disorders by triggering oxidative stress and neuroinflammation.

## 1. Introduction

COVID-19 (Coronavirus disease-2019) was first characterized in China (City of Wuhan) in 2019. After one month of the outbreak, it was declared a public health emergency on 30th January 2020, and a pandemic on 11 March 2020 due to its rapid transmission and infectiousness [1]. As of 1 August 2020, SARS-CoV-2 had spread widely and rapidly in more than 200 countries around the world, causing 17 million cases and a high death toll of 0.68 million people [2]. Globally, as of 19 August 2021, there have been 209,201,939 confirmed cases of COVID-19, including 4,390,467 deaths, reported to the World Health Organization (WHO) (https://covid19.who.int/; accessed on 12 November 2021). Previously, the WHO has expected the mortality rate of COVID-19 to be approximately 4% [3,4,5,6,7]. After that, in 2021, India faced the most devastating situation due to mutation in the virus and the rapid spread of the disease. As of 13 August 2021, the total death rate in India was 429,679, according to the Indian government information (https://www.mygov.in/covid-19; accessed on 12 November 2021).

The coronavirus is a single-stranded RNA belonging to the family of coronaviridae, which infects birds and mammals. In humans, they trigger respiratory infections, and the symptoms are similar to the common cold [8]. The current human infections caused by coronavirus have shown lethal and life-threatening symptoms, including the Severe Acute Respiratory Syndrome (SARS) and Middle East Respiratory Syndrome (MERS) endemics, caused by a zoonotic coronavirus, which is a member of the genus Betacoronavirus. Similarly, the first case of MERS was reported from the kingdom of Saudi Arabia in 2012. After that, 2494 cases of the disease were found, where 858 deaths were recorded, and the fatality rate was 34.4% [9,10,11,12,13,14,15,16,17,18]. Like the previously occurring SARS-CoV and MERS-CoV, the recent SARS-CoV-2 was also caused by a beta coronavirus.

The SARS-CoV-2 has a genome size of ~30 kilobases that encrypt multiple structural and non-structural proteins. They have different proteins, the envelope (E) protein and the nucleocapsid (N) protein, including the spike (S) protein, and the membrane (M) protein [19,20,21,22,23,24]. The severe acute respiratory syndrome-coronavirus-2 (SARS-CoV-2) propagates mainly via respiratory droplets and from person-to-person, getting entry through the respiratory system and lungs, and transmitted to other persons via respiratory droplets, feces, urine, and sweating [25]. The infection spreads to the body from the lungs by activating the body’s immune system, and through the blood circulatory system. After the virus enters the human body, it binds to specific receptors known as Angiotensin Converting Enzyme-2 (ACE2) receptors. SARS-CoV-2 can infect the intestinal mucosal cells, epithelial cells of the tubules of the kidneys, renal tubular epithelial cells, and immune cells. SARS-CoV-2 affects the lower respiratory tract, and causes pneumonia with acute respiratory distress [26,27,28,29]. ACE2 are the entry point for SARS-CoV-2 [7,30,31]. In addition to damage caused by the viral infection, uncontrolled inflammation contributes to the severity of the COVID-19-associated complications [32]. In agreement with this hypothesis, elevated expression of inflammatory cytokines and chemokines has been reported in COVID-19-affected patients [33]. The uncontrolled inflammation occurring in COVID-19, also referred to as cytokine storm, shares similarities with previously reported infections, including SARS-CoV and MERS coronavirus, as comprehensively suggested in the previous studies [34]. Previous studies have suggested that targeted inhibition of IL-6 by Tocilizumab may confer relief [35], although other studies have shown that it is not effective in treating this infection [36].

There are several cytokines which execute the inflammatory cascade, such as tumor necrosis factor-alpha (TNF-α), and it has been suggested that targeted inhibition of TNF-α may reduce the autoimmune inflammatory conditions, indicating that inhibition of TNF-α might be a potential therapeutic strategy to reduce the complications associated with COVID-19 [37]. To combat the inflammation induced by SARS-CoV-2, immunomodulatory agents have gained tremendous interest. As the immunomodulatory agents are the substances that may stimulates or suppress the immune system, and help the body fight infectious disease, monoclonal antibodies, cytokines, and vaccines [38] are rapidly entering into clinical trials, and some are already used in reducing the symptoms associated with COVID-19. The overall studies suggest that inflammation is the main contributor to complications of COVID-19. Similarly, a very recent study published in nature medicine has suggested that the inflammatory cytokine signature may predict the severity and recovery of COVID-19, as the serum IL-6, IL-8, and TNF-α levels were highly associated patient survival [39], indicating that inflammation is the main contributor to complications of COVID-19. Interestingly, these cytokines equally contribute in the pathophysiology of neuroinflammation and neurodegeneration [40]. Apart from the peripheral system, COVID-19 may badly affect the neuronal system by inducing the release of inflammatory cytokines. As suggested previously, neuroinflammation, a host-defense mechanism, is the main factor in the pathophysiology of neurodegenerative conditions, and is linked with the maintenance of normal neuronal structure and functions. A body of evidence from Alzheimer’s disease (AD) and Parkinson’s disease (PD) has suggested activating inflammatory mechanisms, and releasing the inflammatory cytokines in these diseases [41,42]. Microglia, which are the resident innate immune cells, play a significant role in the inflammatory processes in the brain [43]. Moreover, microglia forms the first line of defense for the uncontrolled activation of microglia and neural parenchyma, which affect neurons by releasing several mediators, such as inflammatory cytokines (TNF-α, IL-6, and IL-1β), nitric oxide (NO), prostaglandin E2 (PGE2), and reactive oxidative and nitrosative species [44]. The overall studies showed that inflammation was the main contributor to the pathophysiology of neurodegenerative conditions.

**Figure 1:**
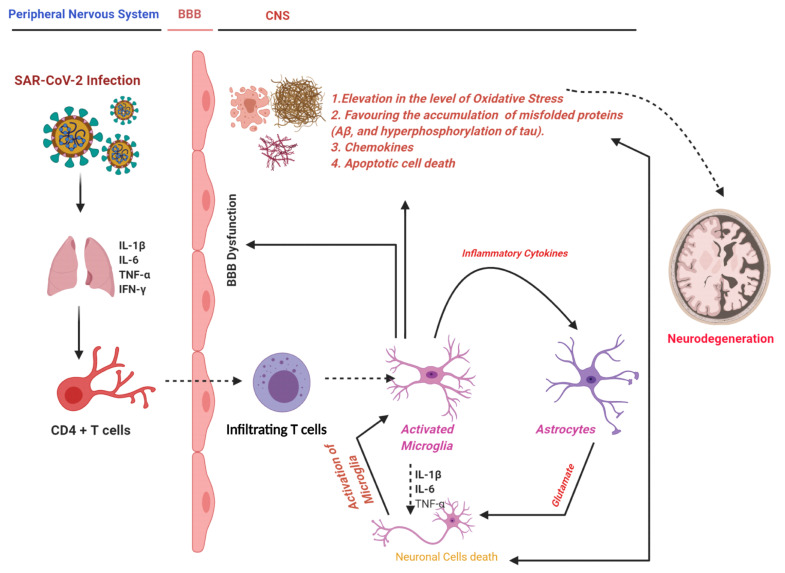
COVID-19 and neurodegeneration.

Recently, several studies have suggested that the COVID-19 aggravates neuroinflammation, and may aid in the pathogenesis of Alzheimer’s disease. There is currently no preventive or curative therapy for the management of COVID-19 and neurodegenerative conditions. In this article, we have collected the current studies conducted on the role of inflammation and oxidative stress in the pathophysiology of COVID-19 and neuroinflammation. We have given the sharing links of the two diseases, the role of the inflammatory mediators, oxidative stress, and other contributors in the pathogenesis of these diseases. We have made insights into the role of the current therapeutic approaches against these diseases, which will enhance our understanding of this disease, and provide insight into its management. Figure 1 shows the COVID-19-associated neural complications, including oxidative, inflammatory mediators, and mitochondrial apoptosis.

A simple sketch showing SAR-CoV-2-induced neurodegeneration through activation of the innate immune system and the oxidative damage, the release of inflammatory cytokines, and mitochondrial apoptosis. IFN-γ: Interferon-gamma; IL-1β: Interleukin-1 beta; Aβ: Amyloid-beta; IL: Interleukin; TNF-α: Tumor necrosis factor-alpha.

## 2. Methodological Approaches

This article is an insight into the resemblance of the two diseases, with the possibility that COVID may induce neurodegeneration. For the compilation of this article, we have collected reputed research articles from different independent databases, such as Google Scholar, ResearchGate, PubMed, and Web of Sciences, covering different aspects of COVID-19 and neuroinflammation, and using the most important keywords: COVID-19, inflammation in COVID-19, inflammation in neurodegenerative diseases, innate immune system activation in COVID-19, oxidative and nitrosative stress in COVID-19, oxidative stress in neurodegenerative diseases, therapeutic approaches against COVID-19, and similarly, many others. The selection of studies was based on the most updated and authentic studies published on the pathophysiology of COVID-19 and neuroinflammation. Reasonable literature has been consulted for the pathological basis of these diseases. We systematically reviewed the available evidence about the pathogenic mechanisms of SARS-CoV-2 infection, the immediate effects of the cytokine storm on the central nervous system, the infiltration of cytokines into the brain, execution of neuroinflammation, and inducers of neuroinflammation. The motivation behind the writing of this review article was to give a comprehensive touch to the shared pathological features of these diseases. Any sort of pre-clinical and clinical studies have been included in our studies. No review papers have been added. The reviewers have created the figures, and no duplicate papers have been added.

## 3. Possible Entry Mechanism of SARS-CoV-2 into the Brains

Several studies have suggested that COVID-19 may exacerbate the AD-like pathological changes in individuals suffering from COVID-19 [45,46]. Here, we have insight into the possibility of induction of AD-like pathological changes in COVID-19-infected individuals.

**Figure 2:**
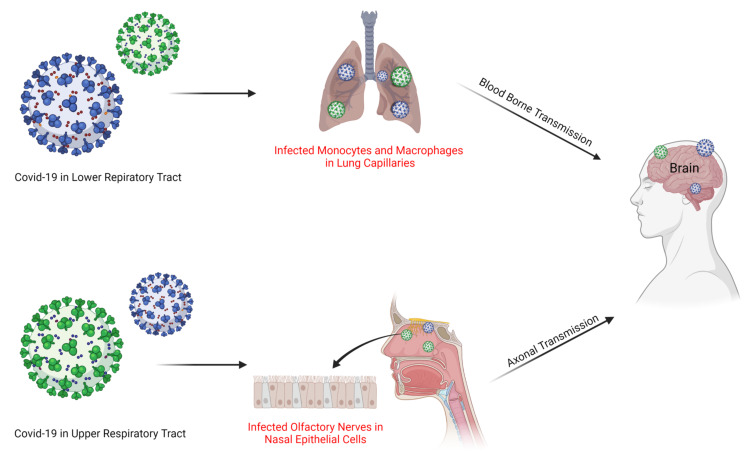
Entry mechanisms of COVID-19 into the brain. The illustration shows the two main mechanisms responsible for the entry of the virus into the brain, i.e., through the lungs (blood born transmission) and the olfactory nerves (axonal transmission) in the epithelial cells.

The brain is a vital organ of the body protected by the blood–brain barrier (BBB), which allows the passage of specific materials to the brain, and prevents the entry of others [47]. As previously described, ACE2 is the cell entry receptor for SARS-CoV-2. Therefore, the presence of this receptor in the central nervous system provides evidence that through ACE2, the SAR-CoV-2 may get entry into the nervous system, and affects the neuronal cells. The expression of ACE2 in brain endothelial cells suggests that SARS-CoV-2 has a higher neuroinvasive ability than other viruses. Evidence supports the concept that SARS-CoV-2 could either directly damage the nerve system, or produce many proinflammatory cytokines, which needs more mechanistic studies [48,49,50,51]. Recently, the nucleic acid of SARS-CoV-2 was detected in the brain tissue and cerebral spinal fluid (CSF) of patients dying from COVID-19. Blood and neuronal pathways are considered the two critical routes for SARS-CoV-2 through which it could get entry into the brain. The blood route for SARS-CoV-2 still needs more verification because of the existence of BBB. However, the neuronal pathway route is considered convenient and well-reported. It is believed that neurotropic viruses such as SARS-CoV-2 first infect the nerve ending, and then migrate to the brain via the brain pathway route. The olfactory tract (olfactory bulb and olfactory nerves) provides an important channel for SARS-CoV-2, usually in the early stages when it infects the respiratory system. The brainstem is considered the operating center of the human body because it controls blood pressure, heartbeat, and respiration. Studies have shown that after infection, SARS-CoV-2 stays for 3-4 days in the upper respiratory tract, and then migrates to the lungs, from where it can invade the brainstem via a synapse-connected route. These findings are crucial to highlight the therapeutic target against the COVID-associated complications, as that respiratory failure may be due to pulmonary lesions or because of the brainstem infection. It has been reported that in COVID-19 patients, the symptoms of intracranial infection (epilepsy, confusion, and headache) appear before the pulmonary infection symptoms (dyspnea, cough, and fever). Therefore, it is essential to do a lumbar puncture (SARS-CoV-2 nucleic acid test on cerebral spinal fluid) and brain magnetic resonance imaging (MRI) tests before diagnosing other symptoms [52,53,54,55,56]. In Figure 2, we have given short illustrations showing the entry mechanisms of the virus into the brain.

## 4. Role of Oxidative Stress in COVID-19 and Neurodegenerative Diseases

As discussed previously, cytokines have been the leading players in the execution of the COVID-19-associated distress, following the same pathway of activation of the innate immune system and pattern recognition receptors, such as Toll-like receptors (TLR4, TLR3, TLR7, and TLR8), and the nucleotide-binding oligomerization domain (NOD)-like receptor family members in lung epithelial cells, retinoic acid-inducible gene-I, dendritic cells, and macrophages [57]. Current studies suggest that the innate immune response in COVID-19 follows the same mechanistic approach as in neurodegenerative diseases [58]. As inflammasome is a significant factor in the cytokine storm, and reactive oxygen species (ROS) is a direct activator of NOD-like receptors P3 (NLRP3) inflammasome [59]. Moreover, the activated TLR and NLR ligands trigger the nuclear factor kappa B (NF-κB)-driven transcriptional activation of NLRP3. Moreover, oxidative stress is also involved in the nuclear translocation of NF-κB, showing that there may be a critical sharing of pathological features in both diseases [60]. An extensively highlighted target of natural flavonoids is transcription factor nuclear factor-erythroid factor 2-related factor 2 (Nrf2), encoded by the *NFE2L2* gene [43,61]. Nrf2 regulates 250 genes involved in the cellular antioxidant system, homeostasis, detoxification of enzymes, transport of drugs, and several cytoprotective effects. The targeted genes of Nrf2, which are associated with cellular defense mechanisms, include antioxidant response elements (ARE), made of antioxidant enzymes (glutamate-cysteine ligase; GCL), drug-metabolizing genes (cytochrome P450s, glutathione S-transferases; GSTs), molecular chaperones, and DNA repair enzymes [62].

**Figure 3:**
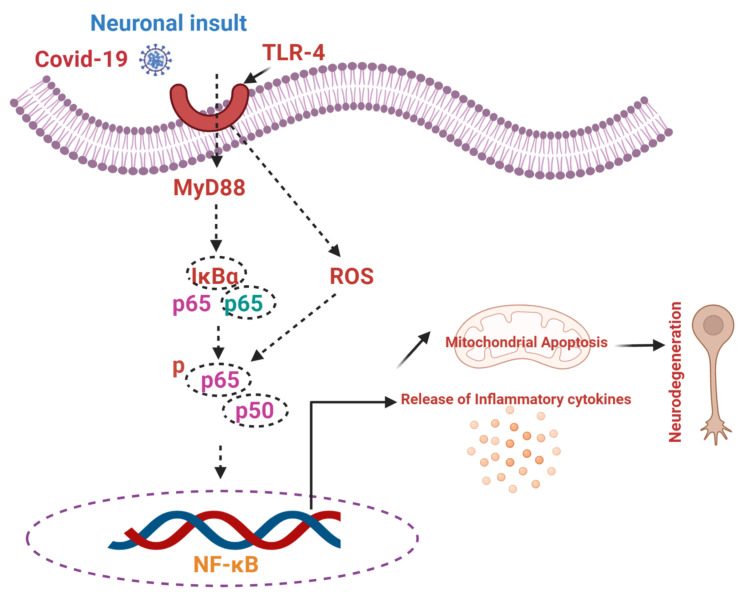
Role of TLR4 and NF-κB in the execution of inflammation, apoptotic cell death, and neurodegeneration. ROS: reactive oxygen species; TLR-4: toll-like receptor-4; ROS: reactive oxygen species; NF-κB: nuclear factor kappa B.

Usually, Nrf2 exists in the cytoplasm, associated with negative regulatory protein, Kelch-like ECH-associated protein-1 (Keap-1), interacting with Nrf2 and working as an adaptor protein, maintaining Nrf2 in an inactive state. During the diseased conditions, Keap-1 senses the oxidative stress via the conjugation of redox-sensitive cysteine residues (Cys151, Cys273, Cys288), and Nrf2 is released from Keap-1 [63]. The dissociation of Nrf-2 from Keap-1 prevents its ubiquitination, increasing its half-life. After the translocation of Nrf2 to the nucleus, it creates a complex with coactivators, and binds to the promoter region (AREs). This binding induces the transcription of cytoprotective genes [64]. Moreover, activation of Nrf2 improves the innate immune system, and regulates inflammatory signaling [65,66]. Figure 3 shows the role of COVID-19 in activating the innate immune system, and executing inflammation and apoptotic cell death.

## 5. Oxidative Stress, Nrf-2, and Proteases Expression in COVID-19 and Neurodegenerative Conditions

Regulated proteolysis is necessary for viral infections, including propagation of SARS-CoV. In the lung, the expression of transmembrane protease, serine 22 (TMPRSS2), human airway trypsin-like protease (HAT), and secretory leukocyte proteinase inhibitor (SLPI) are required to separate the viral hemagglutinin surface protein, and permit the viral fusion and subsequent entry into the host cells [67]. Studies have indicated that oxidative stress and inflammation change the levels of proteases [68]. In the case of influenza, the viral entry and following replication is linked with a reduced expression of Nrf2, and mediated by the activation of proteases in the host [69]. Similarly, the expression of SLPI was increased in Nrf2-deficient mice, increasing inflammation, and showing a balance between oxidative stress and protease expression [70]. Another study has suggested that the reduced expression of Nrf2 induces oxidative stress, and triggers the serine protease, causing hemagglutinin cleavage, and augmenting virion entry into the host cell.

Moreover, activation of Nrf-2 with potent Nrf-2 activators stopped the viral infection, thus stopping viral entry and replication [69]. The collective findings support the notion that activation of Nrf-2 protects the cells from a viral infection by reducing oxidative stress, and regulating protease activities and inflammatory cytokines. Several phytonutrients, such as carotenoids such as flavonoids, lutein, coumarins, isoflavones, indoles, and lignans, have shown protective effects against COVID-19, possibly by regulating the expression of Nrf2 and its associated genes.

Likewise, previous studies have suggested a significant reduction in the expression of Nrf-2 and its associated genes (heme oxygenase-1 (HO-1)) in neurodegenerative conditions, and regulation of Nrf-2 (by the implication of natural or synthetic compounds), have conferred neuroprotection to the mice brains [43,71]. Similarly, specific activators of Nrf2, such as dimethyl fumarate (DMF) and sulforaphane, have shown significant antioxidant, anti-inflammatory, and neuroprotective effects in neurodegenerative conditions [72,73].

Recently, several studies have suggested that potential antioxidant compounds may counteract the severity of COVID-19, which is based on much evidence. As the polyphenolic compounds curcumin could bind to the targeted receptors, including SARS-CoV-2 protease, the receptor-binding domain (RBD) of spike glycoprotein, and the protease domain of ACE has confirmed by molecular docking studies, suggesting that curcumin bind to the SARS-CoV-2 target receptor, and may reduce the severity of the disease [74].

## 6. COVID-19, Inflammation, and Neurodegenerative Diseases

Besides the peripheral effects, COVID-19 may affect the central nervous system (CNS), as MRI studies have shown transient changes in the olfactory bulb associated with COVID-19-related anosmia, highlighting the threat that COVID may affect the brain. A study on rats has shown the intracerebral distribution of gold nanoparticles after inhalation exposure, suggesting that the nanoparticle may be found in nuclei connected to the olfactory and limbic systems, including the hippocampus and cortex olfactory bulb, striatum, entorhinal cortex, and septum [75,76]. Inflammation is a body immune response to infections and tissue injury, known by several inflammatory reactions, including vasodilation and activation of immune cells and plasma proteins to the infected site [77]. Naturally, inflammation is beneficial to the host, and can be handled at a specific time. However, the dysregulated inflammatory conditions may cause severe tissue damage, causing chronic and acute inflammatory conditions [78]. Clinical findings during the outbreaks of coronaviruses—SARS-CoV-2—convincingly suggest that, in addition to virus propagation, the host inflammatory response is a crucial determinant of disease outcome [79].

It has been suggested that COVID-19 patients show an elevated expression of inflammatory cytokines, such as serum interleukin-10 (IL-10), IL-6, and IL-1β, compared to those with less severe symptoms, indicating the role of cytokine storm in the intensity of disease symptoms [80]. Cytokine storm shows an extravagant immune reaction identified by uncontrolled production of inflammatory mediators, cytokines, and chemokines such as TNF-α, interferon-gamma (IFN-γ), IL-1β, IL-6, IL-8, and IL-18 [81]. Physiologically, an adequate release of inflammatory cytokines is essential for the immune system to protect the body against the invasion of viral and other pathogens. However, excessive activation and aberrant immune system response may cause organ injury and damage. The current clinical findings have confirmed the direct correlation of cytokine storm and disease severity in hospitalized COVID-19 patients [81]. This uncontrolled immune response may cause severe pulmonary damage, reduce the lung’s capacity, and induce functional impairment [57]. The damage caused by the COVID-19 to the interstitial arteriolar walls of the lungs shows that severe inflammatory reaction plays a critical role in the pathogenesis of the disease, despite the pathogenic role of the virus [82]. A body of literature has suggested that inflammation is a cardinal feature of COVID-19, which aids in the pathogenesis of the severity of this disease.

**Figure 4:**
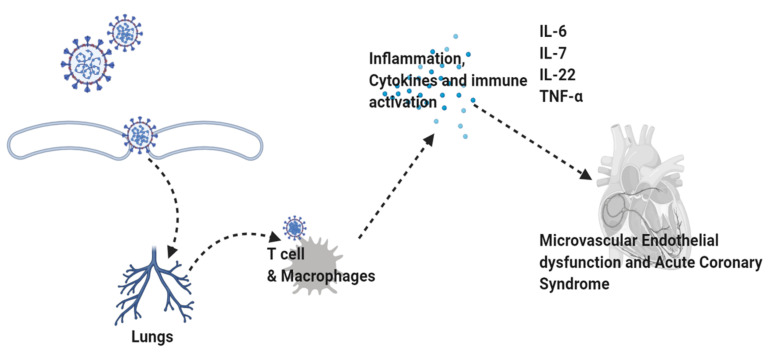
Inflammation in COVID-19. The figure shows the role of macrophages and T cells in the execution of inflammation in COVID-19 and its complications. IL: interleukin; TNFα: tumor necrosis factor alpha.

Recently, several concerns have been raised regarding the impact of COVID-19 on brain health and its consequences as the cardinal features of neurodegenerative diseases through the release of inflammatory cytokines and activation of the immune response [43]. Studies on the role of inflammation in AD, PD, and multiple sclerosis (MS) have identified the involvement of innate immune systems in the initiation of neurodegenerative diseases [41,83]. As the activation of the innate system accelerates the disease progression by releasing the inflammatory mediators, that is why it may serve as a prominent therapeutic target against these diseases [84]. The resident immune cells, such as microglia and brain parenchyma macrophages, are central to the inflammatory processes. In normal conditions, microglia are present in an inactive state compared to other macrophages. However, any change in the microenvironment induces microglial activation, followed by alterations in its morphology and adaptations to several functions, such as phagocytosis and the release of inflammatory cytokines [85]. Moreover, the reactive astrocytes contribute to the process of neuroinflammation by restricting the lesion area, and inducing the release of inflammatory cytokines, and the phenomenon is referred to as ‘neuroinflammation’ [86]. Figure 4 shows the COVID-19 in inflammation.

## 7. COVID-19, NF-κB, and Neurodegenerative Conditions

NF-κB plays a key role in the execution of neuroinflammation and AD-related conditions, a complex protein present in an inactive state in the cytoplasm with its inhibitory proteins, IκBs [87]. Upon activation, the phosphorylation of IκBs causes nuclear translocation of NF-κB, causing the activation of several transcription factors involved in host defense, inflammation, proliferation, and apoptosis [88]. Several factors are involved in the induction of NF-κB, such as bacterial lipopolysaccharides (LPS), ionizing radiation, ROS, and cytokines such as TNF-α, IL-1β, viral DNA, and RNA [89]. The activated NF-κB promotes the activation of a wide range of inflammatory mediators, such as IL-1, IL-2, IL-12, and TNF-α. Moreover, NF-κB has a role in the release of chemokines (such as IL-8, monocyte chemoattractant protein-1 (MCP1), and regulated upon activation, normal T cell expressed, and presumably secreted (RANTES)), adhesion molecules (intercellular adhesion molecule (ICAM), vascular cell adhesion molecule (VCAM), and E-selectin), acute phase proteins (serum amyloid A (SAA)), inducible nitric oxide synthase (iNOS), and cyclooxygenase-2 (COX-2) [89]. Thus, NF-κB serves as a primary transcription factor that regulates various cellular signaling, such as activation of innate immune response to infection, and is related to chronic inflammatory conditions, viral infections, septic shock syndrome, and multi-organ failure [90]. Several studies have suggested that in neurodegenerative conditions, there is an activation of NF-κB, which may further facilitate the neuroinflammatory cascades by activating the cytokines and chemokines [43,91]. Inhibition of NF-κB has shown rescuing effects against different models of neurodegeneration, supporting the notion that NF-κB has the main role in the execution of neuroinflammation [87,92]. Another feature of AD-associated neurodegeneration is mitochondrial apoptosis, and studies have suggested that inhibition of NF-κB may rescue the brain against apoptotic and mitochondrial dysfunction [93]. Also, the inhibition of NF-κB has shown promising rescue effects against synaptic and memory dysfunctions in mice [94]. Misfolding of protein is another feature of AD-associated neurodegenerative conditions, and a study conducted on NF-κB has suggested that activation of NF-κB may activate the beta-site APP cleaving enzyme 1 (BACE-1) activity, thereby triggering the accumulation of amyloid-beta (Aβ). Inhibition of NF-κB may reverse this process [95]. The overall findings have suggested that NF-κB has a critical role in executing neurodegeneration and cognitive dysfunction in AD.

Besides its role in the execution of neuroinflammation and neurodegeneration, NF-κB has shown a significant role in corona-associated infections. A study conducted on SARS-CoV (which was responsible for the outbreak of SARS in 2003) showed that the SARS-CoV nucleocapsid protein triggers NF-κB in cells (Vero E6) [96]. In parallel, SARS-CoV lacking the envelope gene (SARS-CoV-ΔE) showed a reduced level of proinflammatory mediators, neutrophils, reduced lung pathology, and enhanced mice survival [97,98]. The study conducted by DeDiego and his colleagues in 2014 suggested that inhibition of NF-κB enhanced the cell survival rate in vivo and in vitro mice studies, and ultimately reduced lung infection [97]. In vitro studies suggested that the spike (S) protein tempts a cytokine storm in infected cells via NF-κB, which was initiated through activation of TLR in a protein kinase C (PKC)-dependent manner, and may be reversed by the inhibition of NF-κB [99]. Activated NF-κB has a direct effect on the activation of mitogen-activated protein kinases (MAPKs), such as c-jun N-terminal kinase (JNK), p-38, and extracellular signal-regulated kinase (ERK). Furthermore, NF-κB activation also triggers the release of IL, thereby inducing the release of inflammatory cytokines [100]. Besides the direct effects of NF-κB on inflammatory cytokines, the NF-κB, in collaboration with Janus kinase (JAK) and signal transduction and activator of transcription factor 3 (STAT3), contributes to the activation of IL-6. The activation of JAK-STAT induces the release of IFN-γ and cytokines [101]. The above findings support the notion that NF-κB has a prominent role in the pathophysiology of AD and COVID-associated infections equally.

## 8. COVID-19, TNFα, and Neurodegenerative Conditions

One of the cellular factors synthesized by microglial cells in the brain is TNF-α, a pro-inflammatory cytokine and activator of the immune system and resting microglial/astrocytic cells in the brain [102]. In the brain, TNF-α exerts a homeostatic effect by regulating several physiological functions, including sleep, neurogenesis, immune surveillance, and synaptogenesis [103,104]. During the inflammatory conditions, the microglia produces TNF-α and IFN-γ [105], causing enhanced expression of adhesion/costimulatory molecules, such as the major histocompatibility complex (MHC) class II, to maintain the antigen-dependent T-cell activation [106]. The microglia are activated by a set of inflammatory stimuli, including MAPKs (p38, JNK, and ERK1/2) and phospho-NF-κB (p-NF-κB) [107,108]. Besides the execution of the inflammatory processes, TNF-α has a role in processing apoptotic cell death and synaptic and memory dysfunctions [109,110]. A study conducted on TNF-α and IFN-γ has suggested that activation of these cytokines induces the accumulation of Aβ and phosphorylated-tau (p-Tau), which are the cardinal features of AD [102,111]. The above studies have suggested that TNF-α in close association with other inflammatory genes induces neuroinflammation and neurodegeneration.

**Figure 5:**
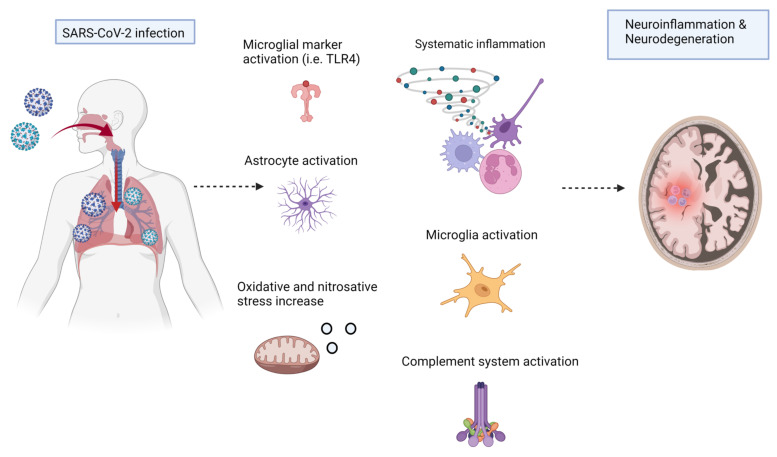
Shared pathological features of COVID-19 and neurodegeneration. The figure, showing the main pathological features of COVID-19 and neuroinflammation, consisted of activating the innate immune system, activation of microglial and astrocytic cells, activation of the complement system, and release of the inflammatory cytokines. TLR: toll-like receptor.

Like neuroinflammation, TNF-*α* has a prominent role in coronavirus-associated complications. Recently, several studies have suggested that targeted inhibition of TNF-α (by monoclonal antibodies (anti-TNF-α), such as certolizumab pegol (CZP) and infliximab (IFX), is a standard therapy for autoimmune diseases [112]. However, it has been ineffective in more than 40% of patients, and is responsible for an increased risk of infection [113,114]. In Figure 5, we have shortly given the overlapping pathological features of COVID-19 and neuroinflammation.

## 9. COVID-19, mTOR, and Neurodegenerative Conditions

Mammalian target of rapamycin (mTOR) signaling regulates several cellular processes, such as metabolism, protein synthesis, apoptosis, transcription, cell cycle, endolysosomes, autophagy, and immune regulation [115,116], and is involved in several pathological conditions, such as inflammation, cancer, and cardiovascular and metabolic diseases [117,118]. Furthermore, different viruses can hijack the mTOR signaling system for completion of viral replication, including influenza [119] and human immunodeficient virus-1 (HIV-1) [120], as well as the coronaviruses MERS-CoV [121,122] and SARS-CoV-2 [123,124]. Blocking the mTOR signaling pathway may reduce the infection and replication of viruses by inducing autophagy, and inhibiting viral protein synthesis [125,126], suggesting that mTOR might be targeted to inhibit SARS-CoV-2 infection and COVID-19 [127]. Several studies have supported this hypothesis that using natural and semisynthetic compounds inhibiting the mTOR signaling is beneficial in suppressing the COVID-19-related complications. For example, polyamines are generated endogenously from arginine and ornithine, used as components of various plants [128,129]. The ingestion of polyamines rescued against age-related cognitive dysfunction [130,131].

Moreover, polyamines confer antioxidant and anti-inflammatory effects by regulating autophagy [132,133,134]. Moreover, the polyamines (spermidine and spermine) upregulate the 5′AMP-activated protein kinase (AMPK), and suppresses the mTOR, thereby inducing autophagy, and suppressing inflammatory dendritic cells. Furthermore, these polyamines have been used against SARS-CoV-2 infection, and appeared to do so by inducing viral degradation in endolysosomes [135].

Resveratrol is a polyphenol enriched in peanuts, berries, and red grapes with potential antioxidant and anti-inflammatory effects in several diseases, such as neurodegenerative diseases, diabetes mellitus, cancer, cardiac diseases, and microbial pathogenesis [136,137]. Studies have suggested that resveratrol enhances autophagy, and kills the cancerous cells by regulating the phosphoinositide 3-kinase (PI3K)/Akt/mTOR and AMPK/sirtuin (SIRT1) signaling [138,139]. Ellen Ter et al. have suggested that co-administration of resveratrol and pterostilbene, the primary antioxidant component of blueberries, potentially reduce SARS-CoV-2 infection in vitro [140]. Similarly, other potential anti-inflammatory agents, i.e., indomethacin, have been shown to reduce canine coronavirus levels in experimental models, and to confer antiviral activity in combination with resveratrol, having a proven tracked record of potent antioxidant activities [141].

## 10. COVID-19, and Accumulation of Misfolded Proteins

The main features of AD are an accumulation of intracellular neurofibrillary tangles and extracellular deposition of Aβ [42]. As suggested, cytokine storm aggravates the pathophysiology of COVID-19; conversely, anti-inflammatory drugs and steroids have shown promising relieving effects against COVID-19 complications, showing that inflammation is a critical feature of COVID-19 [142,143]. Similarly, in neuroinflammation, it has been suggested that elevated oxidative stress and release of the inflammatory cytokines may drive the pathophysiology of AD by inducing the accumulation of misfolded proteins, including Aβ and hyperphosphorylated tau. From a molecular point of view, it is hypothesized that inflammation and oxidative stress has an impact on the misfolding of proteins (Aβ, p-tau) and AD pathology, as suggested previously [144]. In the case of COVID-19-driven inflammatory conditions, it has not yet been elucidated whether the cytokine storm induces misfolding of proteins or not, which needs further studies and exploration.

## 11. Antioxidants and Their Effects against Neuroinflammation

Aging is a normal process associated with alterations in physiological, biological, environmental, behavioral, psychological, and social processes [145]. Oxidative stress is an imbalance between the pro- and anti-oxidant species, which may cause cellular and molecular degeneration [146]. ROS are produced in the biological system to regulate several cellular and molecular functions, including stress regulation, cell survival, inflammation, and autophagy [147,148].

Several studies have suggested that elevation in oxidative stress may be involved in age-related neurodegeneration, although ROS may not be an essential factor for this process [149]. ROS may exacerbate the aging progress via oxidative damage, and affect mitochondrial quality control [150]. Recently, extensive studies have been conducted on the role of antioxidants in the management of neurodegenerative conditions [41,42], which highlighted the importance of these natural compounds in the management of neurodegenerative conditions. Natural compounds have shown rescuing effects against neurodegenerative conditions by several well-known mechanisms [83]. One of the reputed mechanisms is boosting the endogenous ROS regulators, such as Nrf2 and its associated genes. As mentioned before, Nrf2 is a key oxidative stress sensor that induces the transcription of several cytoprotective genes, protecting the cells from excessive oxidative damage [151]. Normally, in the absence of any cellular stressor, Nrf2 is associated with Kelch-like ECH-associated protein 1 (Keap1) in the cytoplasm. When ROS level overcomes the endogenous antioxidant capacity, Keap1 is dissociated from Nrf2, then translocates into the nucleus, where it binds the antioxidant response elements in association with other transcription factors accessory elements. This induces the key antioxidants and cytoprotective genes responsible for regulating oxidative stress, such as glutathione (GSH) synthesis. Recent studies have suggested that specific activation of Nrf2 may confer neuroprotection against a wide range of neurological diseases [152,153] by reducing oxidative stress, microglial-cell-mediated-neuroinflammation, aggregation of Aβ, and mitochondrial apoptosis [154,155]. Several natural compounds, such as fisetin [66], gintonin [41], and hesperetin [43], have shown promising beneficial effects by boosting the expression of Nrf2 and its associated genes [43]. Therefore, based on aforementioned evidence, natural antioxidants have potential neuroprotective effects against neurological diseases.

**Figure 6:**
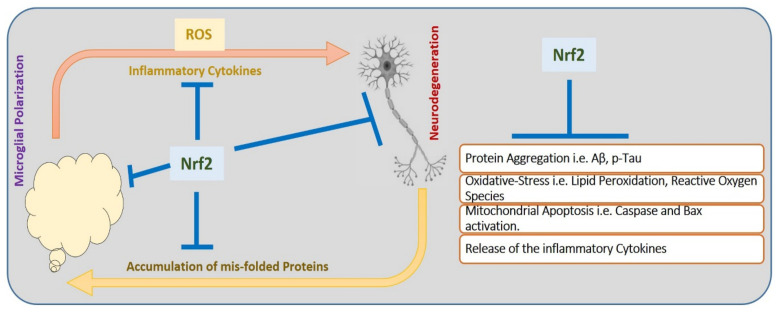
Nrf2 and its role against neurodegeneration. Nrf2 plays a key role against the elevated oxidative stress, neuroinflammation, and mitochondrial apoptosis in neuronal cells. Nrf2: nuclear factor-erythroid factor 2-related factor 2; ROS: reactive oxygen species; Aβ: amyloid beta.

Likewise, in COVID-19, th157ere is a significant increase in ROS, which further aggravates the consequences of the disease. Interestingly, natural antioxidant compounds have drawn much attention in the management of COVID-like neuroinflammation. Few of the drugs that have shown similar protective effects against COVID-19 and neuroinflammation are given here. Figure 6 illustrates the modulatory role of Nrf2 against neurodegeneration.

## 12. Sulforaphane against Neurodegeneration and COVID-19

Sulforaphane is a potent Nrf2 inducer responsible for the induction of cellular defense mechanisms against injury or oxidative insult [156]. Sulforaphane is a product of glucoraphanin (4-(methylsulfonyl) butyl glucosinolate), one of the glucosinolates obtained from cruciferous vegetables, and the most abundant in Brussel sprouts and broccoli [157]. Sulforaphane-enriched fraction has strong protective effects against neuroinflammation, as suggested by its inhibitory effects against the activated microglial cells, suppression of MAPKs, inhibition of NF-κB signaling pathway, and the secretion of inflammatory mediators (iNOS, COX-2, TNF-α, IL-6, IL-1β, PGE2, etc.) [158].

Erden Eren has suggested that sulforaphane reduces the LPS-induced cytotoxicity, neuroinflammation, oxidative stress, miR-155 expression, and switches to Mox phenotype via activation of ERK1/2 and Nrf2/ARE in murine microglial cells [159]. Lalita Subedi has shown the anti-inflammatory effects of sulforaphane against the LPS-activated microglial cells. They have suggested that these effects are conferred via inhibition of p-JNK/AP-1/p-NF-κB, and up-regulates the expression of Nrf2/HO-1 (as master antioxidant regulators) [160]. The findings indicate that sulforaphane has neuroprotective effects against neuroinflammation.

On the other hand, sulforaphane has shown rescuing effects against COVID-19-driven cytokine storm by reducing the oxidative damage, and suppressing the inflammatory mediators. Similarly, Nrf2 induces the expression of a battery of cytoprotective genes, thereby conferring relief against COVID-19 complications [156]. The findings suggest that sulforaphane may act as a double-edged sword against COVID-19 and neuroinflammation.

## 13. Chloroquine and Hydroxychloroquine

**Figure 7:**
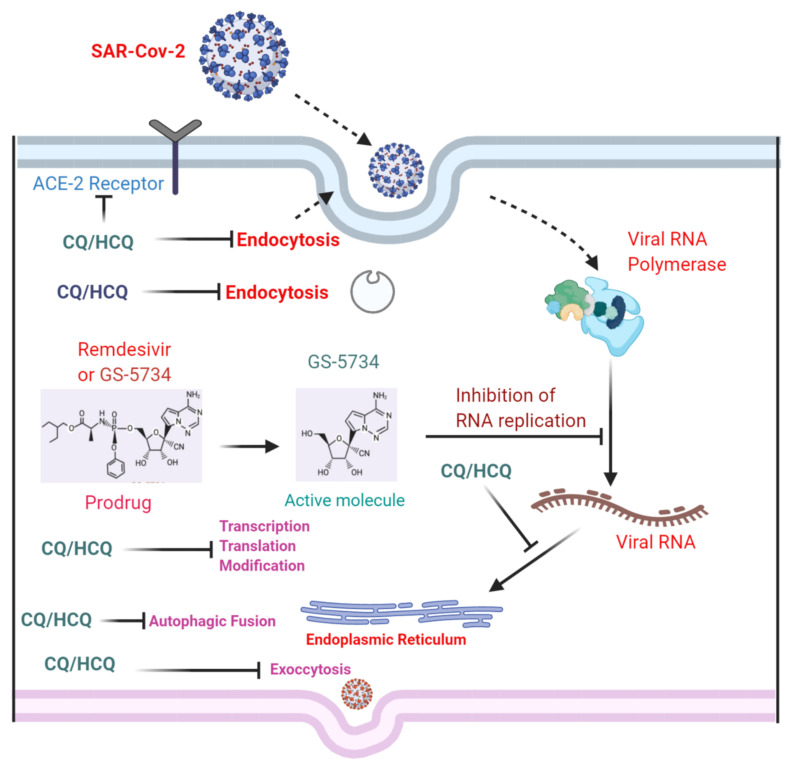
Effects of chloroquine and hydroxychloroquine against COVID-19. A simple diagram showing the protective mechanisms of chloroquine and hydroxychloroquine against COVID-19-associated complications. ACE: angiotensin-converting enzyme; CQ: chloroquine; HCQ: hydroxychloroquine; RNA: ribonucleic acid.

Chloroquine (CQ) and its analog, hydroxychloroquine (HCQ), have been used worldwide as frontline drugs against malarial infection, and also as a prophylactic agent against these diseases [161]. CQ and HCQ have a broad-spectrum antiviral profile against different sorts of viral infections. These drugs are known to block viral diseases by up-regulating the pH at the endosome level, and are essential for cell fusion and interfering with the glycosylation of SARS-CoV cell surface receptors [162]. Currently, there are no specific treatment strategies available for SARS-CoV-2 infection. Several clinical trials are underway against COVID-19-associated complications [163]. Two of the known drugs are CQ and HCQ at different stages of the disease. Based on studies conducted on the usage of these compounds in China, they were reported to pronounce beneficial effects against the coronavirus infection [164,165,166,167,168]. In the case of SARS-CoV-2 infection, the body cells pick up the virus through a receptor, then the virus gets entry into the cell, and attaches to the lysosome. Acidification is necessary for the fusion of lysosome-to-lysosome and the activation of SARS-CoV-2-containing endosome. A lysosome is essentially an acidified organelle in the cell, which may acidify through the proton-pumping V-type ATPase (V-ATPase). V-ATPase pumps the hydrogen ions (H^+^) or essential protons to the lysosome to become acidified. The acidified lysosome fuses with the endosome, which acts as a carrier of coronavirus, and CQ acts as an inhibitor of V-ATPase, inhibiting the proton entry to the lysosome, and preventing it from acidification. Therefore, it blocks the fusion of SARS-CoV-2-containing endosome with the lysosome (Figure 7) [165,169]. Moreover, CQ reduces the glycosylation of the cellular ACE2 receptors, which interferes with SARS-CoV binding to the cell receptor [170]. Although several studies have suggested that CQ and HCQ are effective against the disease, several controversies exist in using these compounds against COVID-19 [171].

Similar to COVID-19-related inflammation, CQ has shown rescuing effects against brain neuroinflammation by suppressing the inflammatory cytokines, such as TNF-α, IL-6, and IL-12, in LPS-challenged mice [172]. A study conducted by Yupeng Long and colleagues suggested that CQ suppresses the TLR4, and activates the macrophages by regulating miR-669n-regulated SUMO-specific protease 6 (SENP6) protein translation, thereby regulating the release of inflammatory cytokines and inflammatory reaction [173]. Glutamate has been used to develop the model of neurodegeneration by inducing metabolic stress. A study conducted on the role of CQ against glutamate-induced neurodegeneration has suggested that CQ regulates the oxidative stress and protein in mouse hippocampal cells through the sigma-1 receptor (S1R) [174]. S1R is expressed in neurons, astrocytes, and microglia, and its activation elicits significant neuroprotection, and endorses neuronal survival via multiple mechanisms, including promoting mitochondrial homeostasis, suppressing oxidative stress, and regulating immune functions [175]. Collectively, studies have suggested that CQ and HCQ have pronounced anti-corona and anti-brain neurodegenerative effects by regulating multiple genes. Here, we have given Figure 7, showing the role of CQ and HCQ in managing SAR-Cov-2-associated complications.

## 14. Melatonin against COVID-19 and Neurodegeneration

Currently, tremendous research has been focused on the pathophysiology of COVID-19, for which different drugs have been investigated. One of them is melatonin, which has shown its effects against COVID-19 by regulating several pathological aspects of corona. Melatonin (N-acetyl-5-methoxytryptamine) is a bioactive molecule (hormone) [176] secreted by the pineal gland, which plays a significant role in the normal physiological functions, mainly the sleep–wake cycle, of animals and humans [177]. Several studies have suggested a wide range of pharmacological and physiological functions of melatonin [178,179]. Melatonin has shown several properties, including antioxidant, anti-inflammatory, anti-excitatory, and immunoregulation [180,181].

Melatonin exerts its effects via receptor-dependent and receptor-independent mechanisms [182], as there are two types of melatonin receptors, MT1 and MT2. MT1 receptor modulates mammalian brain functions, and is distributed in the skin, retina, liver, and hypothalamus [183], whereas MT2 receptor regulates circadian rhythms, and is expressed in the retina, vessels of extremities, and osteoblasts [184]. Melatonin penetrates the cells, and executes several functions by interacting with intracellular and cell surface receptors or scavenging free radicals [180], and as such, modulates several cellular actions, including DNA repairing and cell-to-cell signaling and cellular metabolism [185].

Previous studies have suggested that melatonin modulates the respiratory syncytial virus (RVS) through suppression of TLR-3 signaling, activation of NF-κB, interferon regulatory factor 3 (IRF-3), and reducing the expression of various inflammatory factors. The findings have suggested that melatonin in a dose-dependent and independent manner regulates the expression of TLR-3-induced gene expression in macrophages infected by RSV; repression of NF-κB activity by melatonin seems to be the strategic event leading to the reduction of the expression of inflammatory genes [186]. Furthermore, a study conducted by Huang and colleagues suggested that administration of melatonin significantly reduced the level of malondialdehyde (MDA) and nitric oxide (NO), and enhanced the glutathione (GSH) and superoxide dismutase (SOD) expression in RSV-infected mice. Moreover, melatonin reduced the pro-inflammatory cytokines in the serum of RSV-infected animals, showing regulatory effects of melatonin against RSV-mediated lung injury via suppressing oxidative stress and inflammatory cytokines [187].

In the case of the COVID-19, there are several rationales which suggest that melatonin may reduce the COVID-19 -associated complications, as melatonin has been suggested to block the nuclear translocation of NF-κB, the expression of c-Fos, and suppression of matrix metalloproteinases-3 (MMP-3), which are responsible for pro-fibrotic and pro-inflammatory cytokine production [188,189]. Moreover, it has been suggested that melatonin reduces pulmonary hypertension, which is associated with its potent antioxidant, anti-fibrotic, and vasodilating effects [190]. A study on melatonin has shown the protective effects of melatonin against acute lung injury in pulmonary tissues [191]. 5-hydroxy-2′-isobutyl-streptochlorin (HIS), an analog of melatonin, has shown remarkable anti-inflammatory effects, and has inhibited the entry of immune cells into the lung, thereby reducing the release of inflammatory cytokines (TNF-α and IL-6), which are mediated by the regulation of IFN-β and TLR [191]. Moreover, HIS inhibited the secretion of IL-1β by suppressing the activation of the NLRP3 inflammasome [192]. Lipid peroxidation of the lung surfactant occurs due to the production of ROS from the activated phagocytes, which causes acute lung injury. On its own or in combination with other drugs, melatonin reduces lipid peroxidation of the pulmonary surfactant [193]. All such studies are truly justifying the use of melatonin in the management of COVID-19-associated complications.

Similar to the effects of melatonin against COVID-19 complications, melatonin has shown promising regulating effects against neurodegenerative diseases associated with oxidative stress, neuroinflammation, and mitochondrial dysfunction. Several pieces of evidence show the neuroprotective effects of melatonin against animal models of neurodegeneration. Our group has suggested that melatonin inhibits the scopolamine-induced oxidative stress, JNK activation, regulates the expression of AKT/ERK/cAMP response element-binding protein (CREB) signaling, enhances cell survival and proliferation, and regulates synaptic and memory functions in mice [194].

**Figure 8:**
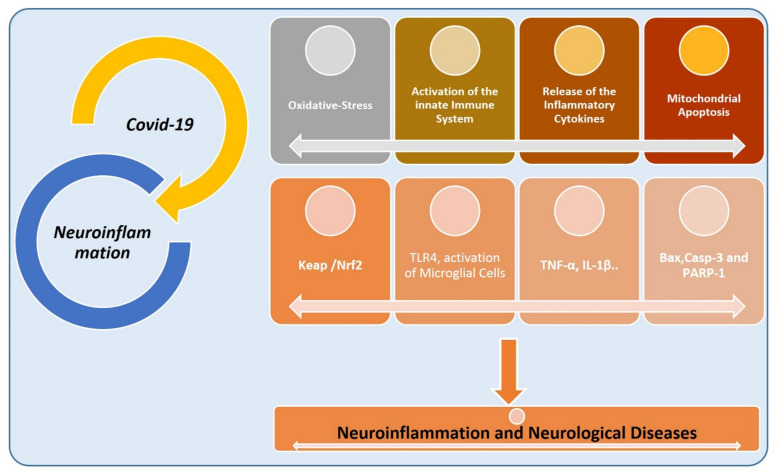
COVID-19 and neuroinflammation.

A study conducted on melatonin against D-galactose induced mouse model of neurodegeneration has suggested that melatonin reduces the D-galactose-induced elevated ROS, and suppresses the receptor for advanced glycation end-products (RAGE). Furthermore, melatonin significantly reduced D-galactose-induced neuroinflammation via suppression of the microglia (Iba-1), astrocytes (GFAP), and other inflammatory effectors, such as p-Iκκb, p-NF-κB65, COX2, IL-1b, NOS2, and TNF-α. In addition, melatonin regulated the apoptotic factors, such as cytochrome C, caspase-3, caspase-9, and poly (ADP-ribose) polymerase 1 (PARP-1) [195]. Apart from these, several studies suggest that melatonin has significant neuroprotective effects against LPS-induced [196] and ethanol-induced [197] neurodegeneration. Overall, findings suggest that melatonin has pronounced neuroprotective effects against the AD-like pathological changes in mice’s brains. Figure 8 shows the COVID-mediated neuroinflammation and its consequences.

The figure illustrates the execution of neuroinflammation by the coronavirus, unveils the role of oxidative stress, activation of the innate immune system, the release of the inflammatory cytokines, and apoptotic cell death.

## 15. Conclusions, Gaps, and Future Perspectives

Herein, we have given the neuroscientific perspective of COVID-19, overlapping pathological features of AD and COVID-19, and shared therapeutic approaches. Furthermore, we have discussed several features of COVID and neuroinflammation, such as elevated oxidative stress, the release of the inflammatory cytokines, and activation of the innate immune system, which equally contribute to the pathology of both diseases [198]. Mechanistically, we have studied the transcription factors involved in the pathophysiology of the two diseases, covering the transcription factor Nrf2 and its associated genes. We have shown elevated oxidative stress, suppression of endogenous antioxidant system, lipid peroxidation, and elevated formation of ROS as the common characteristics of oxidative damage in both of the diseases. Similarly, we have shown that TNF-α and NF-κB are equally involved in the pathophysiology of COVID-19 and neuroinflammation.

Interestingly, several natural and synthetic compounds have shown promising rescuing effects against COVID and neuroinflammation, such as resveratrol, sulforaphane, and melatonin. Additionally, the present study recommends the use of anti-inflammatory and anti-oxidant drugs against COVID-19-associated complications. Moreover, the in silico studies conducted on COVID-19 suggested that cannabivarin (CVN) and cannabidiol (CBD) obtained from cannabis can bind to the ACE2, interleukin-6, and transmembrane serine protease, which are significantly involved in post-COVID-19 complications [199]. Similarly, another in silico study has suggested that amongst the reported molecules used against viral infection, HIV protease inhibitors and RNA-dependent RNA polymerase inhibitors showed binding to COVID-19 enzyme. Moreover, methisazone (protein synthesis inhibitor) and CGP42112A (angiotensin AT2 receptor agonist) might be proven as the best therapeutic option against COVID-19 complications [200]. One important point which has not been included in the current manuscript is the mutations in coronavirus, and its effects on neurodegenerative conditions. Similarly, we have not added the effects of natural and synthetic compounds against the mutant strains of coronavirus.

More rigorous preclinical and clinical studies are warranted to unveil the effects of COVID-19 on the accumulation of misfolded protein and AD-like pathological changes in COVID-19-infected individuals, which will unveil the effects of COVID-19 on brain health and neuronal functioning.

## Data Availability

Not applicable.
